# Genetics of Parkinson's Disease and Other Diseases of the Extrapyramidal System

**DOI:** 10.2174/138920291501140306110937

**Published:** 2014-02

**Authors:** Jolanta Dorszewska

Advancements in science and technology in the second half of the twentieth century have led to increased life expectancy, which has contributed to the increased number of cases typical for old age diseases, including Parkinson's disease. Estimates suggest that, in the aging population, the number of patients with Parkinson’s disease will maintain an upward trend. 

Parkinson's disease is a disorder of the extrapyramidal system. Other diseases causing extrapyramidal disorders, with the exception of Parkinson’s disease, are called atypical parkinsonism or parkinsonism *plus*. These diseases include: multiple system atrophy, MSA; progressive supranuclear palsy, PSP; corticobasal degeneration, CBD; dementia with Lewy bodies, DLB. Their diagnosis, especially in the initial stage of the disease, is not clear.

Parkinson's disease is one of the most common degenerative diseases of the central nervous system. It is known that the intravital diagnosis of Parkinson's disease is difficult. Certain diagnosis of this disease is only possible after *postmortem* neuropathological examination of the brain for lesions typical for this disorder. 

Lack of early diagnosis of Parkinson's disease can make it difficult to treat effectively, contributing to the progression of the disease, and may further lead to reduced quality of life for these patients. Since the 1960s, the primary drug used to treat Parkinson's disease is L-dopa. Unfortunately, the effectiveness of L-dopa therapy in patients with Parkinson’s disease often decreases with progression of the disease and is associated with the appearance of numerous side effects. Side effects of L-dopa treatment include: dyskinesia, atherosclerosis, depression and dementia.

Around the world, research is underway to find an effective therapy for patients with Parkinson's disease, including drugs that are neuroprotective and inhibit the degenerative process within the *substantia nigra* in the midbrain. At the same time, it appears that elucidating the currently unknown pathological processes of this disease may lead to more effective diagnosis and pharmacotherapy. 

Currently, it is believed that genetic study may be an essential element of intravital diagnosis of patients with Parkinson's disease and the elucidation of the mechanism of interaction between the genes associated with the pathogenesis of this disease probably helps explain unfamiliar pathways of selective damage to dopaminergic neurons in Parkinson's disease patients.

